# Prevalence and associated risk factors of *Cryptosporidium* infection in calves and hospitalized humans in Libo Kemkem, North Western Ethiopia

**DOI:** 10.1002/vms3.70040

**Published:** 2024-09-17

**Authors:** Habtamu Tamrat, Yemane Tekle, Mussie Hailemelekot, Negus Belayneh

**Affiliations:** ^1^ School of Animal Science and Veterinary Medicine College of Agriculture and Environmental Sciences Bahir Dar University Bahir Dar Ethiopia; ^2^ Datan Agro Processing Private Limited Company; ^3^ Andasa Livestock Research Center Bahir Dar Ethiopia

**Keywords:** calf, *Cryptosporidium*, human, Libo Kemkem District, prevalence, risk factors

## Abstract

**Background:**

*Cryptosporidium* infection is one of the major causes of acute gastroenteritis and diarrhoea caused by a protozoan parasite affecting vertebrates and humans. The disease is prevalent in cases of immunocompromised individuals. Despite the impact of the diseases in calf and hospitalized humans, well‐documented studies are not available in the study area.

**Objectives:**

The objectives of this study were to determine the prevalence of *Cryptosporidium* infection in calves and hospitalized humans and assess the major associated risk factors associated with *Cryptosporidium* infection in calves and hospitalized humans.

**Method:**

A cross‐sectional study was conducted from November 2020 to March 2021 on calf and human *Cryptosporidium* infection in Libo Kemkem District, North West Ethiopia. A total of 193 calves and 122 human stool samples admitted to the hospital were used for this study. Three kebeles were selected purposely, and individual calves were selected using a simple random sampling method. A number of sampled calves were allocated proportionally to the selected kebeles. Human samples were collected using a systematic random sampling method. Faecal and stool samples were examined using a modified Ziehl–Neelsen staining method.

**Result:**

The overall prevalence of calf and human *Cryptosporidium* infection found in this study was 15.5% and 11.5%, respectively. Age of calf, breed, body condition, water source, faecal consistency and hygienic condition were found significantly (*p* < 0.05) associated with *Cryptosporidium* infection in the calf. Similarly, the source of potable water, immunocompromisation and contact with domestic animals were found to be significantly (*p *< 0.05) associated with *Cryptosporidium* infection in humans.

**Conclusion:**

There was a higher prevalence of *Cryptosporidium* infection in calves and humans in Libo Kemkem District. Therefore, the implementation of proper prevention methods of zoonotic *Cryptosporidium* infection between calf and human beings through significant risk factors is mandatory. Furthermore, additional studies to investigate the levels of economic importance of the disease should be conducted.

## Graphical abstract

A cross‐sectional study was conducted on calf and human *Cryptosporidium* infection in Libo Kemkem District, North West Ethiopia, with the aims of determining the prevalence of *Cryptosporidium* infection in calf and from hospitalized human and assessing the major associated risk factors associated with the infection in calves and hospitalized human. The overall prevalence of calf and human *Cryptosporidium* infection found in this study was 15.5% and 11.5%, respectively. Age of calf, breed, body condition, water source, faecal consistency and hygienic condition were found significantly (*p* < 0.05) associated with *Cryptosporidium* infection in the calf. Similarly, the source of potable water, immunocompromisation and contact with domestic animals were found significantly (*p* < 0.05) associated with *Cryptosporidium* infection in humans. The prevalence of *Cryptosporidium* infection in calves and humans was higher in Libo Kemkem District, North West Ethiopia. Therefore, the proper implementation of prevention methods for the *Cryptosporidium* infection is needed to mitigate risk factors for *Cryptosporidium* infection with a view of reducing the prevalence of *Cryptosporidium* infection in calf and human.

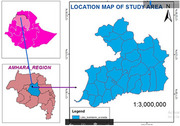

## 1 INTRODUCTION

*Cryptosporidium* infection is one of the most common gastrointestinal diseases in a wide spectrum of vertebrates, including humans (Chalmers & Katzer, 2013; Xiao & Fayer, 2008). *Cryptosporidium* is a protozoan parasite in the phylum Apicomplexa (Radostitis et al., 2007). Currently, 26 *Cryptosporidium* species have been recognized. Of which, *Cryptosporidium hominis* and *Cryptosporidium parvum* were reported to infect humans (Adamu et al., 2014; Bouzid et al., 2013; Lebbad et al., 2013). *C. parvum* infects many different hosts, including cattle, swine, horses and small animals (Radostitis et al., 2007). *C. parvum* has two distinct genotypes known as human genotype 1 and bovine genotype 2. Both genotypes are capable of causing disease in humans. Farm animals are not commonly infected with Genotype 1, although recently infections have been experimentally produced in lambs and piglets and mixed infections with Genotypes 1 and 2 have been observed in calves (Radostitis et al., 2007).

*C. parvum* is a common cause of infection in young ruminants and is found in many species of mammals, including humans. It is considered a significant cause of varying degrees of naturally occurring diarrhoea in neonatal farm animals (Radostitis et al., 2007). Cryptosporidiosis is an emerging protozoan disease caused by *Cryptosporidium* species that causes gastrointestinal infection in humans, cattle, sheep, goat, pig and horse worldwide (Ayana & Alemu, 2015).

Once detection of infection occurred as early as 5 days of age, the greatest intensity of excretion of the organism in the faeces of individual calves occurred between 9 and 14 days. Infected calves develop resistance to reinfection with lower probabilities of oocyst excretion in older and adult cattle (Radostitis et al., 2007). Most commonly, the agent acts in concert with other enteropathogens to produce intestinal damage and diarrhoea (Radostitis et al., 2007).

*Cryptosporidium* infection is one of the zoonotic diseases that have recently emerged (Adamu et al., 2014; Szonyi et al., 2010; Zaidah et al., 2008). Human infections are mostly caused by highly host adapted *C. hominis* and *C. parvum*. Eight *Cryptosporidium* species have been less frequently or sporadically detected in humans (De Lucio et al., 2016). The routes of transmission could be person to person through direct or indirect contact, animal to animal, animal to human, water‐borne (drinking or recreational water) and food‐borne (Painter et al., 2015). Infection in healthy individuals is usually self‐limiting and resolves within 2 up to 3 weeks of profuse, watery, non‐bloody diarrhoea, weight loss, abdominal pain, anorexia, fatigue and cramps (Warren & Guerrant, 2007). In immunocompromised persons, infection is more serious and can cause prolonged, debilitating, life‐threatening illness (Chalmers & Davies, 2010). Owing to the lack of prophylactic and therapeutic measures against the disease, the mortality rate in humans is an emerging public health issue worldwide (Kothavade, 2011). Because specific therapy or vaccine for the control of this parasite is not yet available, preventing infection depends on avoiding exposure to the parasite and maintaining immune competence (Adamu et al., 2014).

In Ethiopia, few studies on *Cryptosporidium* infection were conducted on dairy farms in the Central and Southern part of the country and reported prevalence ranging from 2.3% to 27.8% (Abebe et al., 2008; Adamu et al., 2010, 2014; Regassa et al., 2013; Wegayehu et al., 2013, 2016). Similarly, few studies carried out on human immunodeficiency virus patients showed prevalence ranging from 12.1% to 43.9% (Adamu & Petros, 2009; Dijs‐Elsinga et al., 2010; Mariam et al., 2008). The prevalence in normal, non‐diarrhoeal children was reported to be between 8.1% and 12.2% (Ayalew et al., 2008; Tigabu et al., 2010) and that of diarrhoeal children and adults was between 5.6% and 9% (Adamu et al., 2010).

Few studies conducted in Ethiopia have shown the occurrence of *Cryptosporidium* infection in both cattle and humans with different levels of prevalence. In Ethiopia, in general and in the study area in particular, there are different risk factors associated with the occurrence of the disease. Such as a wide range of close human contact with domestic animals and their excreta, use of common water points for animals and humans, annually high flood coverage from grass lands towards water points, mismanaged disposal of animal and human waste, use of common animal and human house, raw milk consumption and poor hygienic conditions may contribute to the transmission of the disease from animal to human and vice versa. Furthermore, as Ethiopia is a wider country, there was no study conducted in and around Libo Kemkem District. Therefore, this study was initiated to determine the prevalence of *Cryptosporidium* infection in calves and in hospitalized humans and to investigate the major risk factors associated with *Cryptosporidium* infection in calves and hospitalized humans in Libo Kemkem District, North Western Ethiopia.

## 2 MATERIALS AND METHODS

### 2.1 Study area

**FIGURE 1:**
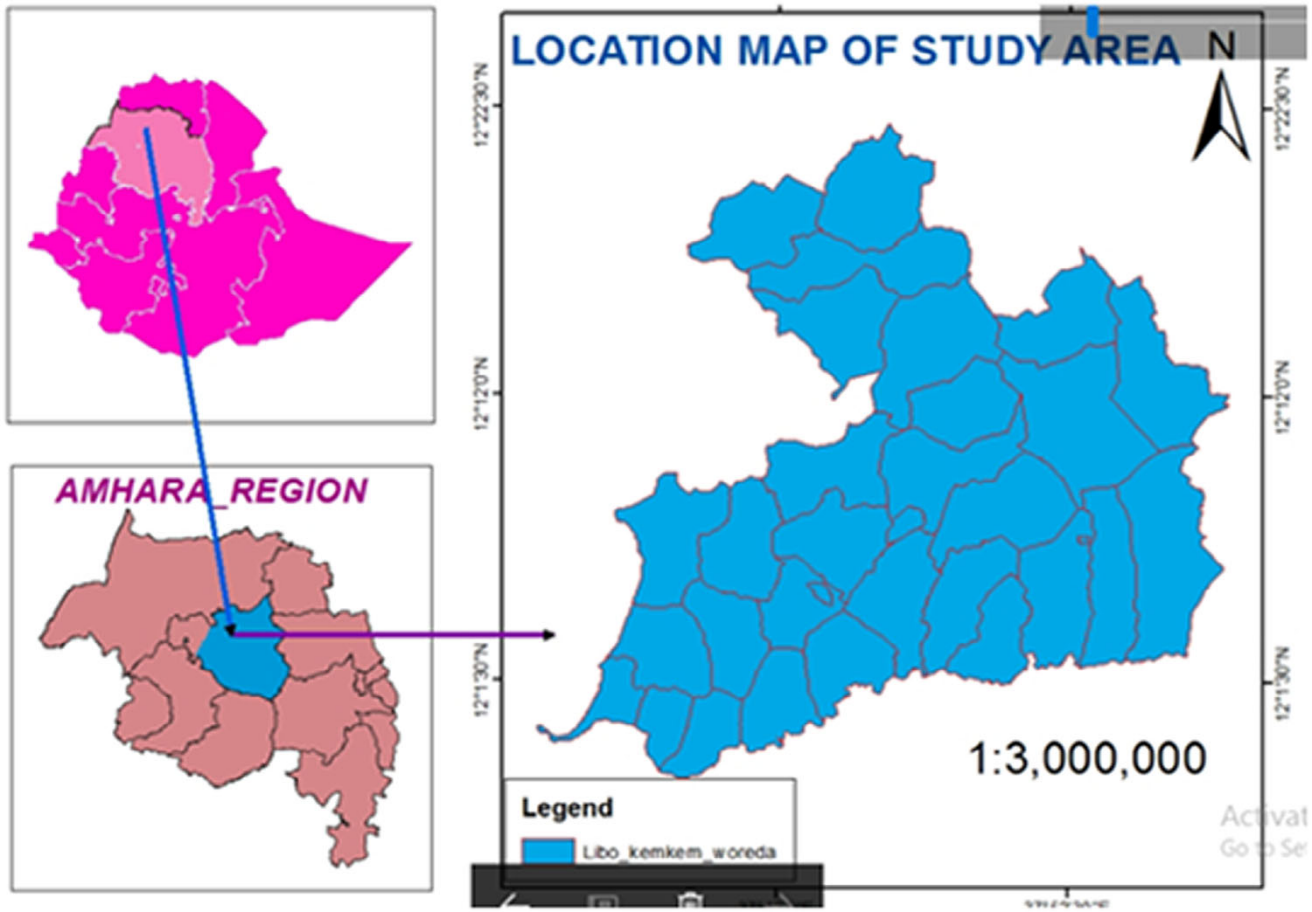
Map of the study area.

The study was conducted in Libo Kemkem District, which is 746 km North West of Addis Ababa, the capital city of Ethiopia. It has an area of 1560 km^2^ and a total population of 198,374, of which 100,951 are males and 97,423 are females. It is located at 11°57′46.6″–12°25′32.6″ N latitude and 37°34′48.9″–38°3′30.9″ E longitudes. The altitude of the district ranges from 1800 to 3000 m above sea level, and the temperature ranges from 18 to 25°C. The area receives annual rainfall ranging from 900 to 1400 mm. From the total area of land, 51% is cultivable, 8.3% is pasture, 5.9% is forest or shrub land, 17.98% is covered with water and the remaining 17.03% is considered degraded or other. The livestock populations of the district were estimated to be 123,007 cattle, 19,248 sheep, 38,483 goats, 33,434 equines, 76,819 poultry and 13,721 beehives (Central Statistical Agency, 2019/2020). The maps of the study area are displayed in Figure 1.

### 2.2 Study design

A cross‐sectional study design was conducted from November 2020 to March 2021. Calves of age groups ranging from 1 week up to 12 months and hospitalized humans of all age groups were considered in this study. Risk factors to the occurrence of *Cryptosporidium* infection like age category (1 week to 6 months and greater than 6–12 months), sex, breed (local and cross), body condition (poor, medium and good), source of water, management system (semi‐intensive and extensive), hygienic practice (poor, medium and good), geographic location (lowland, midland and highland) and faecal consistency (diarrhoeal and non‐diarrhoeal) of calves were considered. Likewise, the occurrence of the disease in hospitalized humans was determined using age, sex, source of drinking water, contact with domestic animals, immunocompetence and stool consistency as important risk factors.

### 2.3 Sampling procedure and sample size determination

Libo Kemkem District had 32 kebeles, which are agro‐ecologically categorized as 6 highlands, 20 midlands and 6 lowlands. Three kebeles were selected purposely representing the three agro‐ecological locations. The sampling method used was simple random sampling. A total of 193 faecal samples were collected from local and cross breed calves from selected kebeles proportionally. For human samples, 122 stool samples were taken from individuals who were admitted and intended to perform a stool examination by medical personnel at Libo Kemkem hospital by a systematic random sampling method in consecutive 16 days. Where an individual enrolled their names in the first 8 consecutive odd numbers were sampled. (That was eight samples for a consecutive 15 days daily and the remaining two samples on the last 16th day.) Sample size was calculated using the study of Thrusfield (2005) with 18.6% expected prevalence of calf cryptosporidiosis and 9% hospitalized human cryptosporidiosis at 95% confidence interval 5% absolute precision. Hence, a total of 193 calves and 122 hospitalized humans were used for this study.

### 2.4 Faecal sample collection

Before collecting the faecal samples, informed consent was obtained from calf owners. Fresh faecal samples were collected directly from rectums of the study animals using sterile disposable plastic gloves. Each sample was collected using a labelled separate universal bottle. The samples were transported in a cool box to Bahir Dar Animal Health Investigation and Diagnostic Laboratory on the same day of collection and were appropriately preserved at refrigeration temperature until processed within 48 h of arrival. At the time of sampling, the name of the farm (owner), date of sampling, consistency of the faeces (diarrhoeal, soft or normal), age, sex, address, management system (semi‐intensive and intensive) and, body condition (poor, medium, good) of the calves were recorded.

### 2.5 Stool sample collection

Prior to stool sample collection, each patient was interviewed to collect important information on the disease. Accordingly, all methods were carried out in accordance with guidelines and regulations of the International Centre for the Ethics of Research involving humans. Every individual invited to sampling was asked whether they were exposed to immunocompromising diseases in their life or not. These data were used to estimate the levels of *Cryptosporidium* oocyst between immunocompromised and non‐immunocompromised individuals. A labelled disposable screw‐capped plastic bag was given to each patient to bring stool samples. Once the samples were collected, preservation was made by adding 3–5 mL of potassium chromate (Salman et al., 2015). Finally, stool samples were kept in a cool box and transported to the laboratory.

### 2.6 Coproscopical examination

#### 2.6.1 Sheather's floatation technique

Faeces of 3 g were weighted from each animal and mixed with 10 mL of sugar solution. Then it was poured through a tea strainer into a beaker, and then the solution was added into a 12 mL centrifuge tube and placed into the centrifuge. The tube was then filled with sugar solution about 1 in. from the top of the tube without putting coverslips on the tube and centrifuged at 1200 rpm for 5 min. Then the test tube was removed from the centrifuge and filled to the top with sugar solution, then covered with a cover slip on the tube and kept at standing for 10 min. Finally, the cover slip was removed from the tube and was placed on a slide labelled with the animal name or number. The entire cover slips were examined at 40× objectives, and the oocysts were identified, recorded according to standard methods (Kaufmann, 1996).

#### 2.6.2 Modified Ziehl–Neelsen staining

Thin faecal smears were air dried and passed quickly through a flame. The smears were stained with Ziehl–Neelsen's carbol fuchsin solution for 2 min and then rinsed with tap water. The smears were rinsed for a few seconds with acid alcohol (3% hydrochloric acid in 70% ethanol). Again, the smears were rinsed with tap water. The smears were counterstained with brilliant Green (0.5%) for 2 min and rinsed again with tap water. The slides were air‐dried and examined microscopically at 100× objective using oil immersion. *Cryptosporidium* oocysts appeared bright red granules on a blue background. Only those which were positive on the modified Ziehl–Neelsen technique were recognized as positive, and others were registered as negative (Kaufmann, 1996). In calves, faecal samples of at least 1.2 × 105 *Cryptosporidium* oocysts per gram of faeces were stated as cryptosporidium positive (Constable et al., 2017; Medema et al., 2001).

### 2.7 Data management and analysis

All the data collected were entered into Microsoft Excel spreadsheet programme, checked for competence and then analysed using SPSS version 20.0 statistical software. Descriptive analyses like percentages and prevalences were calculated. The association of individual risk factors with an outcome variable was screened by univariate logistic regression. Those variables significantly associated with the outcome variable at 5% or *p* < 0.05 significance level in the univariate analysis were recruited for multivariable logistic regression to see their independent effect. In the multivariable analysis, a model was fitted for each outcome variable by stepwise backward elimination of insignificant variables (*p* > 0.05). Multivariable logistic regression was used to see statistically significant associations among risk factors. Significant level was determined at 95% confidence level and (*p* < 0.05).

## 3 RESULTS

A total of 193 calves (140 local and 53 cross‐breed) and 122 hospitalized humans were examined. The overall prevalence of *Cryptosporidium* infection in calves and hospitalized humans in the district were 15.5% and 11.5%, respectively (Table 1).

**TABLE 1:** Overall prevalence of *Cryptosporidium* infection in calves and hospitalized human.

Risk factors	*N*	Number positive	Prevalence (%)
Calves	193	30	15.5
Human	122	14	11.5
**Total**	315	44	13.97

*Note*: *N* = number of sampled animal.

### 3.1 Risk factors

A total of nine risk variables were tested separately using univariate logistic regressions. Of these, seven potential risk factors were significantly (*p* < 0.05) associated with *Cryptosporidium* infection in calf (Table 2).

**TABLE 2:** Potential risk factors significantly associated with *Cryptosporidium* infection in calf using univariate logistic regression.

Risk factors	Categories	Prevalence (%)	OR	95% CI	*p* value
Age	≤6 months	21.59	1.47	0.65–3.33	0.037
	6–12 months^*^	10.48			
Breed	Cross‐breed	24.53	2.35	1.05–5.28	0.037
	Local^*^	11.4			
Body condition	Poor	26.32	0.27	0.10–0.75	0.012
	Medium	12.5	0.76	0.26–2.26	0.620
	Good^*^	9.38			
Faecal consistency	Diarrhoeal	20.54	0.37	0.15–0.90	0.029
	Non‐diarrhoeal^*^	8.64			
Water source	Spring	13.83	3.35	1.25–8.99	0.016
	River	25.45	2.18	0.86–5.52	0.101
	Tape^*^	9.59			
House hygiene	Poor	24.6	0.38	0.14–1.01	0.049
	Medium	11.77	0.92	0.31–2.71	0.881
	Good^*^	10.94			
Geographic location	Lowland	20.48	0.38	0.12–1.20	0.049
	Midland	13.84	0.61	0.18–2.11	0.432
	Highland^*^	8.88			

*Note*: ^*^ = reference variable.

Abbreviation: OR, odd ratio.

These potential risk factors were further evaluated by a multivariable logistic regression analysis; six variables were significantly associated (*p* < 0.05) with *Cryptosporidium* infection in calves. These include age, breed, body condition, source of water, hygienic practice and faecal consistency (Table 3).

**TABLE 3:** Potential risk factors significantly associated with *Cryptosporidium* infection in calf using multivariable logistic regression.

Risk factors	Categories	Prevalence (%)	OR	95% CI	*p* value
Age	≤6 months	21.59	2.99	1.70–4.92	0.021
	6–12 months^*^	10.48			
Breed	Cross‐breed	24.53	2.70	1.91–12.01	0.049
	Local^*^	11.4			
Body condition	Poor	26.32	0.59	0.08–4.16	0.043
	Medium	12.5	0.15	0.02–1.32	0.243
	Good^*^	9.38			
Faecal consistency	Diarrhoeal	20.54	0.27	0.04–1.92	0.016
	Non‐diarrhoeal^*^	8.64			
Water source	River	25.45	1.16	0.23–6.87	0.027
	Spring	13.83	0.75	0.04–2.35	0.154
	Tape^*^	9.59			
House hygiene	Poor	24.6	1.15	0.55–3.69	0.042
	Medium	11.77	1.27	0.92–4.31	0.804
	Good^*^	10.94			

*Note*: ^*^ = reference variable.

Abbreviation: OR, odd ratio.

Regarding *Cryptosporidium* infection in humans, a total of six variables were tested separately using univariate logistic regression, of which four potential risk factors were significantly (*p* < 0.05) associated with the occurrence of *Cryptosporidium* infection in humans (Table 4).

**TABLE 4:** Potential risk factors associated with *Cryptosporidium* infection in human using univariate logistic regression.

Risk factors	Categories	Prevalence (%)	OR	95% CI	*p* value
Source of water	Tape water^*^	7.58			
	Spring	2.77	4.88	1.43–16.63	0.011
	River	40	10.80	4.25–18.34	0.031
Immunocompromised	Yes	23.9	0.13	0.03–0.45	0.003
	No^*^	3.95			
Stool consistency	Diarrhoeal	21.3	4.80	1.41–16.34	0.012
	Normal^*^	5.33			
Contact with animals	Yes	16.92	0.25	0.07–0.97	0.043
	No^*^	5.26			

*Note*: ^*^ = reference variables.

Abbreviation: OR, odd ratio.

Drinking water, immunocompromisation and contact with domestic animals were statistically significant (*p* < 0.05) risk factors when evaluated with multivariable logistic regression analysis (Table 5).

**TABLE 5:** Potential risk factors associated with *Cryptosporidium* infection in human using multivariable logistic regression.

Risk factors	Categories	Prevalence (%)	OR	95% CI	*p* value
Source of water	Tape water^*^	7.58			
	Spring	2.77	6.16	1.53–26.65	0.024
	River	40	11.44	3.26–20.25	0.048
Immunocompromised	Yes	23.9	0.12	0.03–0.51	0.007
	No^*^	3.95			
Contact with animals	Yes	16.92	0.15	0.06–3.22	0.033
	No^*^	5.26			

*Note*: ^*^ = reference variables.

Abbreviation: OR, odd ratio.

## 4 DISCUSSION

In this study, the overall prevalence of *Cryptosporidium* infection in calves was found to be 15.5%. This is comparable with the reports of Abebe et al. (2008), Ayele et al. (2018), Manyazewal et al. (2018), Terfa and Getushe (2023), and Wegayehu et al. (2016) who reported 15.8%, 18.6%, 18.6%, 17.6% and 17.1% from Central, Western and North West Ethiopia, respectively. Similarly, Geurden et al. (2006) reported 19.2% from Zambia, Lefay et al. (2000) reported 17.9% from France and Xiao and Fayer (2008) reported 11.9% from the USA; all reported a comparable prevalence in dairy calves. The current finding was lower than the report of Regassa et al. (2013), which was 27.8% from Haramaya and 24.0% by Berhanu et al. (2017) from Asella Town. Similarly, Paudyal et al. (2013) reported 58.3% from Nepal, and Nguyen et al. (2007) reported 33.5% in Vietnam. However, the present finding of *Cryptosporidium* oocyst in calves was higher than the results of Wudu (2004), which was 6.7% from Debre Zeit and 4.2% by Berhanu et al. (2017) in and around Asella. This variation among the prevalence might be associated with the geographic difference, study design, diagnostic techniques, production system and management as well as the season of the year when the study was conducted, as supported by Venu et al. (2013). In addition, study reports point out a range of possible sources of calf hood infection, such as contaminated pens, water supplies, buildings, tools and contact surfaces, shoes and clothing of animal handlers as well as flies and birds serving as mechanical vectors (Conn et al., 2007; Manyazewal et al., 2018).

The current result is in line with the report of Ayele et al. (2018), who reported calves less than 6 months old were highly affected (28.4%) than older calves; Kiros et al. (2017), who reported calves under 6 months (24.8%) were at higher risk of infection as compared to the older calves in Asella Town, South Eastern Ethiopia. Similar results were obtained by Hussin et al. (2016), who reported 40% of *Cryptosporidium* infection was detected in calves from Week 1 to 3 months of age than the older calves from Iraq. Comparable age‐related distribution pattern results had been reported by different researchers (Fayer et al., 2006; Feng & Xiao, 2017; Kváč et al., 2006; Liu et al., 2009; Nazemalhosseini‐Mojarad et al., 2011; Plutzer & Karanis, 2007; Silverlås et al., 2010; Thompson et al., 2009), who explained that the animal is becoming resistant with age due to the immune development through time. It was also agreed by Xiao et al. (2004) and Nguyen et al. (2007) that, although *Cryptosporidium* was observed among all age groups, the prevalence of the disease in calves less than 6 months is significantly higher than older cattle.

According to this finding, breed is a statistically significant risk factor (*p *= 0.049). A report in Haramaya, Eastern Ethiopia (Regassa et al., 2013) reported a higher prevalence of *Cryptosporidium* among exotic (28.8%), followed by local (27.3%), and then crosses (25.0%) breeds of calves. On the other hand, Berhanu et al. (2017) reported 38.3%, 24.9% and 10.3% prevalences of *Cryptosporidium* in exotics, followed by cross‐breeds and local breeds of calves, respectively, with a higher significance difference (*p *= 0.002) among the breeds in Asella Town, South Eastern Ethiopia. A study performed in China reported that different breeds of calve had shown significant differences in the prevalence and species of *Cryptosporidium* (Gong et al., 2017).

Taking calves body condition as a risk factor, the obtained prevalence of *Cryptosporidium* in calves with poor, medium and good body conditions was statistically significant (*p* = 0.043). This can be related to a decrease in the immune system (Khalil et al., 2018; Swai & Schoonman, 2010). This result is in line with the reports of Berhanu et al. (2017) and Terfa and Getushe (2023), who reported the higher prevalence of *Cryptosporidium* infection in poor body condition calves done around Asella Town, South Eastern Ethiopia.

In this study, faecal consistency showed a significant association (*p* = 0.016). That is, calves that are diarrhoeal are (20.54%) and non‐diarrhoeal (8.64%). This result is lower than the report of Ayele et al. (2018) and Terfa and Getushe (2023), who noted a higher occurrence of diarrhoeal calves (35.5%) in North Western and Western Ethiopia, respectively. Similarly, Paudyal et al. (2013) have reported 66.7% of the diarrhoeal samples and 28.9% of the non‐diarrhoeal samples were positive from Nepal. Other researchers also indicated the association of *Cryptosporidium* with diarrhoea and mucoid faeces (Del Coco et al., 2008; Díaz‐Lee et al., 2011; Enemark et al., 2002) and stated that clinical diarrhoea was restricted to calves younger than 2 months, in which the highest number of oocysts was detected. Calves infected with *Cryptosporidium* showed more signs of diarrhoea due to the invasion and colonization of the epithelial surface by the parasite, which results in loss of epithelial cells (Chen et al., 2003; Robinson et al., 2003).

In this study, water source was also one of the major risk factors in the prevalence of *Cryptosporidium* oocyst in relation to the source of water. River water (25.45%), followed by spring water (13.85%) and tap water (9.59%), and a statistically significant result is obtained among the sources of water (*p* = 0.027). The current result is in line with the report of Ayele et al. (2018), who reported 25.13% of calves drinking from river water were found more exposed to *Cryptosporidium* infection. Increased risk of *Cryptosporidium* was seen in farms using river/stream water sources; this could be due to exposure of these water sources to stool of human, faeces of domestic and wild animals, which have been contaminated with oocysts of *Cryptosporidium*. River water is heavily contaminated with oocyst of *Cryptosporidium* (Feng et al., 2011).

Calves’ poor hygienic status showed a prevalence of *Cryptosporidium* oocyst 24.6%, which was higher and statistically significant (*p* = 0.042) than the prevalence in medium (11.77%) and good (10.94%) hygiene status. This result is in line with the report of Ayele et al. (2018), who reported a prevalence of *Cryptosporidium* oocyst in poor hygienic status (34.4%) and good (15.2%) hygiene calves (*p* = 0.001) from North West Ethiopia.

In this study, the overall prevalence of *Cryptosporidium* oocyst in hospitalized human was found to be 11.5%. The current study was in line with the reports of Girma et al. (2014) and Terfa and Getushe (2023), who reported (9.56%) from Ambo and Toke Kutaye districts of West Shoa Zone of Oromia and Yirgalem Hospital Ethiopia, respectively. Manyazewal et al. (2018) reported (9%) from Addis Ababa, Ethiopia; Adamu and Petros (2009) reported (8.6%) from Adama, Afar and Dire Dawa, Ethiopia; and Wegayehu et al. (2013) reported (7.3%) and (7.8%) consecutively from North Shewa, Ethiopia. Adamu et al. (2010), who reported (7.6%) from nine regions of Ethiopia; Ali and Ali (2013), who reported (13.6%) from Iraq, Bamaiyi and Redhuan (2016), who reported (12.2%) from South Africa, Yılmazer et al. (2017) who reported (8.93%) from Turkey, Salman et al. (2015) who reported (16.28%) from Iraq and Zuurendonk (2014), who reported (13.40%) from South Africa. However, it was far lower than the reports of Adamu and Petros (2009) who reported (26.9%) from Addis Ababa, Ethiopia, Wegayehu et al. (2016) who reported (18.6%) from Addis Ababa, Ethiopia. Doungmala et al. (2019) who reported (51%) from Iraq, and Sherchand et al. (2016), who reported (29.4%) from Nepal. This result is also higher than the report of De Lucio et al. (2016), who reported (4.6%) from North West Ethiopia; Gebre et al. (2019), who reported (3.3%) from Jimma, South West Ethiopia; Bamaiyi and Redhuan (2016), who reported (5.6%–8%) from South Africa; and Samra et al. (2016), who reported (5.6%) from South Africa.

The study result showed a prevalence of *Cryptosporidium* oocyst 7.58%, 2.77% and 40%, respectively, among people who are using tape water, spring water and river water for drinking purposes. With a statistically significant difference (*p* = 0.024). This result is in agreement with the findings of Mumtaz et al. (2010), who reported 77.8% of the total *Cryptosporidium* infections in children using well water. A similar finding, showing a significant association of *Cryptosporidium* infection with consumption of contaminated water, was reported by Sulaiman et al. (2005) from Kuwait. A statistical significance difference (*p* = 0.007) is also obtained in this study with a prevalence of 3.95% *Cryptosporidium* oocyst between immunocompromised and non‐immunocompromised individuals.

In this study, the prevalence of *Cryptosporidium* oocyst showed a significant difference between those individuals who have and have no contact with domestic animals (*p* = 0.033). This is in agreement with earlier studies of close contact with cattle and their faeces as the major risk factor of *Cryptosporidium* infections in humans (Adamu et al., 2014; Ehsan et al., 2015; Ng et al., 2012; Nuchjangreed et al., 2008; Wegayehu et al., 2013). In a study in Pakistan, the majority of the infected children had a history of contact with animals, and the authors suggested that animals could be reservoirs of human infection (Mumtaz et al., 2010).

## 5 CONCLUSION AND RECOMMENDATIONS

The present study has clearly revealed that there is a high prevalence of *Cryptosporidium* infection in calves and humans in the study area. Age, breed, body condition, drinking water source, faecal consistency and hygienic conditions were statistically significant risk factors for the occurrence of *Cryptosporidium* in calves. In the same way, source of potable water, immunocompromisation and contact with domestic animals were significantly associated with the prevalence of human *Cryptosporidium*. Generally, the current study provided initial baseline data regarding the prevalence and major risk factors of *Cryptosporidium* infection in calves and hospitalized humans. Based on the findings of this study, due attention should be given to disposal of manure and animal wastes, hand washing following contact with animals, use of separated water point sources for animals and humans and uses of separated calf pens apart from human living houses. Additionally, veterinarians and health extension workers should work together to tackle the zoonotic importance of *Cryptosporidium* infection. Further epidemiological investigation at the molecular level should be conducted.

## AUTHOR CONTRIBUTIONS

**Habtamu Tamrat**: Conceptualization; investigation; methodology; writing – original draft preparation; formal analysis; writing – review and editing; supervision; validation. **Yemane Tekle**: Conceptualization; data curation; investigation. **Mussie Hailemelekot**: Formal analysis; writing – review and editing; supervision. **Negus Belayneh**: Methodology; writing – original draft preparation; writing – review and editing.

## CONFLICT OF INTEREST STATEMENT

The authors declare no conflicts of interest.

## FUNDING INFORMATION

No funding was obtained for this study.

## ETHICAL APPROVAL AND CONSENT TO PARTICIPATE

The authors confirm that the ethical policies of the journal, as noted on the journal's author guidelines page, have been adhered to. The study protocol was reviewed and approved by the Ethical Clearance Committee of School of Animal Science and Veterinary Medicine, Bahir Dar University (Ref. No. SASVM/205/2018). Accordingly, all methods were carried out in accordance with guidelines and regulations of the International Centre for the Ethics of Research involving animals and humans. Before conducting the research, animal owners were informed of the objectives and the benefits of the study, and verbal consent was obtained for the collection of faecal samples. The stool samples were collected by strictly following the standard operational procedure and by minimizing any discomfort by health professionals. All subjects provided written informed consent. Any information was kept confidential, and an anonymous test was utilized.

### PEER REVIEW

The peer review history for this article is available at https://publons.com/publon/10.1002/vms3.70040.

## Data Availability

The data for this study are available from the corresponding author upon reasonable request.
